# The COVID-19: Current understanding

**DOI:** 10.14202/vetworld.2020.1998-2005

**Published:** 2020-09-26

**Authors:** Shweta Tripathi, Mayukh Mani Tripathi

**Affiliations:** 1 Department of Home Science, Government PMRS College, Pendra road, Gaurela, Pendra, Marwahi, Chhattisgarh, India; 2 Department of Medical and Health, Community Health Center, Chopan, Sonbhadra, Uttar Pradesh, India

**Keywords:** coronavirus disease 2019, diagnosis, severe acute respiratory syndrome coronavirus-2, treatments

## Abstract

In December 2019, China reported several cases of a new coronavirus disease (COVID-19). The COVID-19 outbreak, which was initially limited to Wuhan, China, has rapidly spread worldwide. Infection of the disease occurs through exposure to the virus through inhalation of respiratory droplets or if a person touches a mucosal surface after touching an object with the virus on it. The common symptoms of COVID-19 are fever, dry cough, dyspnea (difficult or labored breathing), fatigue, chest pain, and myalgia (muscle pain), etc. Real-time polymerase chain reaction is used to detect the virus in sputum, throat, nasal swabs, and secretion of lower respiratory samples. Early diagnosis, isolation, and supportive care are necessary for the treatment of the patients. The present review aims to provide recent information on COVID-19 related to its epidemiology, clinical symptoms, and management. This article also summarizes the current understanding of severe acute respiratory syndrome coronavirus-2 and its history of origin.

## Introduction

On December 31, 2019, the Municipal Health Authority of Wuhan in the Hubei Province of China reported several cases of pneumonia of an unknown etiology. All the patients had a common exposure to the Hunan seafood market [1-3]. On January 7, 2020, the Chinese Center for Disease Control and Prevention discovered a novel coronavirus from a throat swab sample of one of those patients. Initially, the World Health Organization named it 2019 nCoV [1,2], but later on February 11, 2020, it was renamed as severe acute respiratory syndrome coronavirus-2 (SARS-CoV-2) by ICVT, and WHO designated the disease as coronavirus disease 2019 (COVID-19) [4,5]. Before the COVID-19, the two outbreaks of coronavirus are Middle East Respiratory Syndrome Coronavirus (MERS-CoV) and SARS-CoV. Both occurred over the past 20 years, MERS was first detected in Saudi Arabia in 2012 and affected 2494 people with 858 mortalities [6], and SARS was originated from Guangdong (China) in 2003 and resulted in more than 8000 infections and 774 deaths in 37 countries [7].

The COVID-19 outbreak, which was initially limited to Wuhan, has rapidly spread worldwide. As of now, August 15, 2020, 21,056,181 cases, and 765,771 deaths have been reported globally [8]. COVID-19 made its entry into India through various international travels. The first laboratory-confirmed case of COVID-19 was reported from Kerala on January 30, 2020 [9]. Now, it has spread in almost every part of India. According to the Ministry of Health and Family Welfare, currently, there are 677,444 active cases of COVID-19 in India [10].

Researchers from all over the world are working very closely and efficiently on this pandemic [4,5]. As several studies are currently in progress, new revelations are emerging every day. Therefore, this article aims to provide recent information about the COVID-19 and the relation to its epidemiology, clinical symptoms, and treatments. This article also summarizes the current understanding of SARS-CoV-2 and its history of origin.

## History and Origin

COVID-19 causing virus (SARS-CoV-2) belongs to the *Coronaviridae* family under nidovirales order [11]. Further, this family consists of two subfamilies; coronavirinae and torovirinae, and within the coronavirinae, there are four genera: (a) Alpha-coronavirus; this includes the human coronavirus human coronavirus (HCoV)-229E and HCoV-NL63, (b) beta-coronavirus; this contains SARS-HCoV, HCoV-HKV1, and MERS-CoV, (c) gamma-coronavirus; this covers the viruses of Whales and birds, and (d) delta-coronavirus; this includes viruses from pigs and birds [12]. SARS-CoV-2 comes within the beta-coronavirus genus, which also contains two deadly viruses: SARS-CoV and MERS-CoV. Beta-coronavirus genus consists of many subgroups. The SARS-CoV-2, SARS-CoV, and bat SARS-like CoV belong to Sarbecovirus, whereas the MERS-CoV belongs to Merbecovirus[12,13]. SARS-CoV-2 is an enveloped, single-stranded, and positive-sense RNA virus [14]. The size of this virus ranges from 65 to 125 nm in diameter, and the size of single-stranded RNA varies from 26 to 32 kbs [11]. The crown-like spikes on the outer surface of the virus give it the name of corona [14]. Spikes are glycoprotein in nature and responsible for host attachment [15]. Other proteins of coronaviruses include nucleoprotein, membrane proteins, and accessory proteins [16,17].

Beta-coronavirus found in Pangolins and bat coronavirus (RaTG13) isolated from *Rhinolophus affinis* might be the parental viruses responsible for the origin of SARS-CoV-2 [17,18]. Bat coronavirus (RaTG13) shares 96.2% genome sequence identity with SARS-CoV-2 [17]. Despite the genomic similarity, RaTG13 might not be the immediate ancestor of SARS-CoV-2 because of disparity in receptor binding protein domain (unlike the SARS-CoV-2, it does not use the ACE2 receptor of the host cell for entrance). Whereas, virus from pangolins uses the same receptor (ACE2) but differs in the overall genome sequence, thus, ruling out its possibility of being an immediate ancestor of SARS-CoV-2 too. However, the virus isolated from pangolins is closely related to both; SARS-CoV-2 and RaTG13. Therefore, bats might be the original host of SARS-CoV-2 [13].

## Epidemiology

In late December 2019, several residents of Wuhan, the capital of the Hubei Province of China, visited the hospital with complaints of fever, dry cough, fatigue, and infrequent gastrointestinal symptoms [19]. All the cases had a common exposure to a seafood wholesale wet market, the Hunan Wholesale seafood markets [1]. However, the first laboratory-confirmed case of COVID-19 was on December 1, 2019 [19]. On December 31, 2019, China issued an epidemiologic alert and notified WHO about this mysterious disease. On January 1, 2020, authorities shut down the Hunan seafood market, assuming it the source of this virus, as several environmental samples of this market had tested positive for SARS-CoV-2 [20]. However, based on a genomic study, research suggested that the virus came into the market from an unknown location [21].

In China, patients were increasing rapidly, and soon after, a large number of patients started coming into the hospital with no history of exposure to the live market. Reported cases of clusters of families suggested human to human transmission of this virus [1]. Due to heavy transportation load and the massive migration of the Chinese population, during the Chinese New Year festival, several new cases were being reported in many cities and provinces of China. As several tourists had also visited China during this period, they returned to their home country with this deadly virus. Thailand recorded the first exported case of COVID-19 on January 13, 2020 [19]. Soon after, several other countries reported its first confirmed cases.

On January 21, 2020, India started thermal screening of passengers arriving from China at seven airports (Delhi, Mumbai, Kolkata, Chennai, Bangalore, Hyderabad, and Cochin) to fight COVID-19. Later, the government extended thermal screening to up to 20 airports [22]. These efforts did not work as effectively as hoped, and India recorded its first case of COVID-19 of a student from Kerala on January 30, 2020; she had just returned from Wuhan [9]. Soon after, other states reported its first confirmed cases of COVID-19. As numbers of cases were increasing daily, the Govt. of India imposed a nationwide lockdown on March 25, 2020, to control the spread of this disease [23]. Till the date of writing this article (August 15, 2020), a total number of 2526192+65002 laboratory-confirmed cases of COVID-19 from 35 states and Union Territories, out of which 1,915,580 (71.91%) recovered, while 50,924 (1.93%) deaths are reported in India [8,10]. Maharashtra is the worst affected state with a total number of confirmed cases of 584,754 (https://arogya.maharashtra.gov.in/1175/Novel--Corona-Virus seen on August 15, 2020). The frequency of confirmed cases has been increasing rapidly, and India is on the 3^rd^ position in a tally of worst-affected countries globally [8].

Genome analysis of SARS-CoV-2 revealed that the 5’ end of the SARS-CoV-2 genome contains ORF1ab, and this ORF1ab occupies the majority of the genome. ORF1ab encodes for polyprotein pp1ab and which comprises 15 nonstructural proteins. The 3’-end of the genome contains four structural proteins and eight accessory proteins such as 3a, 6, 7, 8, and 10 [24]. Viruses can undergo mutation, and the genome of RNA viruses can mutate 1 million times higher than the host genome. These mutations can be either harmful or beneficial to the virus [25]. Phylogenetic analysis of Indian SARS-CoV-2 isolates suggests that these are strongly related to isolates reported from other parts of the world. Most ORFs are highly conserved, whereas mutations were also identified in some ORFs. The study further revealed that most isolates from India have key mutations at 614^th^ position of the S protein and 84^th^ position of the ORF8, which has been reported to be associated with high virulence and high transmission rate [25].

Soon, it was clear that the infection of COVID-19 occurs through exposure to the virus through inhalation of respiratory droplets or if a person touches a mucosal surface after touching an object with the virus on it [26]. A study reported the presence of SARS-CoV-2 in stools suggesting the possibility of fecal-oral route transmission [27]. Infection through conjunctiva is also possible [28]. Initially, scientists believed that newborns could get an infection from COVID-19 positive mothers. However, a researcher ruled out the intrauterine transmission in its study, where nine newborns did not contract the disease from their mothers [29]. Although all age groups and genders are susceptible to the attack of this virus, mostly male patients of the 25-55 age group have been reported [30,31]. Elderly and people with weak immune function and hepatic dysfunction are more at risk [30].

SARS-CoV-2 is highly infectious, with an effective reproductive number of 2.9 (higher than SARS-CoV-1.77) [32,33]. The mortality rate of COVID-19 is 3.59% globally and 1.93% in India [8]. The lower mortality rate in India might be due to its demographic constitution. However, due to lack of testing and surveillance, there is a possibility that the number of deaths related to COVID- 19 could not be differentiated hence not reported. India reported a 71.91% recovery rate for COVID-19 [10].

## Clinical Symptoms

According to the World Health Organization, COVID-19 affects different people in different ways. On average, it takes 5-6 days from when someone is infected with the virus for symptoms to show; however, it could also possibly take up to 14 days [34]. Most infected people will develop mild-to-moderate illness symptoms and recover without hospitalization. The most common symptoms include fever, dry cough, and tiredness, and less common symptoms are aches and pains, sore throat, diarrhea, conjunctivitis, headache, loss of taste or smell, and a rash on the skin, or discoloration of fingers or toes [34]. Severe symptoms such as difficulty in breathing or shortness of breath, chest pain or pressure, and loss of speech or movement, require immediate medical attention [34].

## Diagnosis

According to the Ministry of Family and Health Welfare of India; a suspected case is defined as a patient with acute respiratory illness (fever and at least one sign/symptom of respiratory disease, e.g., cough, and shortness of breath) and a history of travel to or residence in a location reporting community transmission of COVID-19, 14 days prior of the beginning of symptoms. It is also defined as a patient with an acute respiratory illness and has been in contact with a confirmed or probable COVID-19 case in the past 14 days before symptom onset; ora patient with severe acute respiratory illness (fever and at least one sign/symptom of respiratory disease, e.g., cough, shortness of breath; and requiring hospitalization). In the absence of an alternative diagnosis that thoroughly explains the clinical presentation, a probable case is a suspect case for whom testing for the COVID-19 virus is inconclusive or a suspect case for whom testing could not be performed for any reason, and, a confirmed case is a person with laboratory confirmation of COVID-19 infection, irrespective of clinical signs and symptoms [35]. The definition of a confirmed or suspected case differs from country to country.

The testing strategy in India used for COVID-19 is discussed below [36]; Real-time polymerase chain reaction (RT-PCR) is the gold standard test for detecting cases of COVID-19. The average time taken is around 4-5 h from receipt of a sample to getting the result. The advantage of this platform lies in its accuracy of detection as well as the ability to run up to 90 samples in a single run. Whenever possible, it is advised to use RT-PCR as the frontline test for the diagnosis of SARS-CoV-2.

The TrueNat and CBNAAT systems have also been deployed for the diagnosis of COVID-19 given the availability of customized cartridges. These platforms have a quick turnaround time (30-60 min), but only 1-4 samples can be tested in one run, limiting the maximum numbers that can be tested to 24-48 samples/day only.

Rapid point-of-care antigen detection test (for diagnosis along with RT-PCR): This test is a promising tool for quick diagnosis of SARS-CoV-2 in field settings. The assay is known as Standard Q COVID-19 Ag kit and has been developed by SD Biosensor with a manufacturing unit at Manesar, Gurugram. On validation, the test has been found to have a very high specificity with moderate sensitivity.

IgG antibody test for COVID-19 (only for surveillance and not diagnosis): IgG antibodies generally start appearing after 2 weeks of the onset of infection, once the individual has recovered after infection, and last for several months.

To ramp up testing capacity, ICMR has approved a total of 1000 COVID-19 testing labs in both public (730) and private sector (270). This includes RT-PCR labs (557); TrueNat Labs (363); and CBNAAT Labs (80) [36].

## Treatment

At present, there is no specific treatment available for COVID-19 [37]. Isolation is the first step in managing the virus. Symptomatic supportive care such as oxygen therapy, fluid management, maintenance of vital signs and blood pressure, and treatment of secondary bacterial infection with the antibiotic is recommended [38]. Some victims of COVID-19 developed ARDS and septic shock very rapidly, eventually leading to multiple organ failure [1,2]. Therefore, early recognition of the suspected case and immediate isolation for the containment of disease is necessary [39]. National Institute of Health (NIH), USA, recommended the following guidelines to treat COVID-19 and severe conditions caused by COVID-19 [40].

### Chloroquine and hydroxychloroquine

It is an old anti-malarial drug, which has shown an *in vitro* inhibitory effect on the growth of SARS-CoV-2 [41]. Recent studies from China and France observed that the administration of this drug in COVID-19 patients produced significant results in both clinical outcomes as well as in viral clearance [42,43]. Chloroquine/hydroxychloroquine, when given with Azithromycin, yielded better results [44]. The Govt. of India has also recommended the same combination of these drugs for the management of COVID-19 [35]. However, the USA Food and Drug Administration (FDA) only allows this drug for the treatment of particular adolescent and adult patients hospitalized for COVID-19. Recently, the World Health Organization suspended all its clinical trials as few studies suggested a high mortality risk of this drug. Therefore, more researches are needed to establish the safety and efficacy of this drug.

### Convalescent plasma therapy

This therapy utilizes plasma of COVID-19 recovered patients to treat other patients. Several studies reported a shorter hospital stay and lower mortality rate in SARS patients treated with convalescent plasma than those who were not treated with it [45-47]. Recently a medical practitioner from Shanghai used plasma therapy to treat COVID-19 patients and yielded positive results with rapid recovery, suggesting it could be a potential treatment for COVID-19 patients. However, more clinical trials are needed to prove the safety and effectiveness of convalescent plasma transfusion in SARS-CoV-2 infected patients [48].

### Remdesivir

Researchers developed this antiviral drug to treat Ebola, MERS, and SARS-CoV. It is a monophosphide prodrug that acts as a nucleotide inhibitor and causes premature termination of viral RNA replication [49]. It has also shown interference with the NSP12 polymerase of SARS-CoV-2 in the *in vitro* study [50]. Some studies also reported that remdesivir alone and in combination with chloroquine and beta interferon blocked the SARS-CoV-2 [41,51,52].

### Corticosteroids

Patients with severe COVID-19 can develop a systemic inflammatory response that can lead to lung injury and multisystem organ dysfunction. It has been suggested that the anti-inflammatory effects of corticosteroids might prevent or mitigate these deleterious effects [53]. Therefore, the COVID-19 Treatment Guidelines Panel of the NIH recommends using dexamethasone 6 mg/day for up to 10 days for the treatment of COVID-19 in mechanically ventilated (AI) patients and in those patients who require supplemental oxygen but not the mechanical ventilation (BI) [40].

Apart from these therapies, researches on antiviral drugs and vaccines are in progress which includes:

### Antiviral drugs

Lopinavir and ritonavir are the two most critical potential candidates against SARS-CoV-2. The combination of these two drugs (usually a component of HAART regiment to treat AIDS) has shown an *in vitro* antiviral activity against SARS-CoV-2. However, more randomized trials are mandatory to prove its effectiveness against this novel coronavirus [54].

### Mesenchymal stem cells

MSCs are multipotent adult stem cells that are present in most human tissues, including in the umbilical cord. MSCs can self-renew by dividing and can differentiate into multiple types of tissues, including osteoblasts, chondroblasts, adipocytes, hepatocytes, and others, which has led to a robust clinical research agenda in regenerative medicine [55] It is hypothesized that MSCs could reduce the acute lung injury and inhibit the cell-mediated inflammatory response induced by SARS-CoV-2 [56].

MSCs are investigational products that have been studied extensively for broad clinical applications in regenerative medicine [55] and their immunomodulatory properties [57]. No MSCs are approved by the FDA for the treatment of COVID-19. There are insufficient data to assess the use of MSCs for the treatment of COVID-19. The FDA has recently issued several warnings about patients becoming potentially vulnerable to stem cell treatments that are illegal and potentially harmful [58].

### Tocilizumab

Tocilizumab, a recombinant monoclonal antibody against the interleukin-6 receptor, has been used to mitigate the cytokine release syndrome associated with chimeric antigen receptor T-cell therapy and has been proposed as a potential therapy for the cytokine storm syndrome associated with severe COVID-19 pneumonia based on small phase two studies [59-65]. An observational study reported reduced mortality in ICU requiring COVID-19 patients who had received tocilizumab [66]. The Indian Council of Medical Research has recommended the use of tocilizumab in mechanically ventilated patients not improving despite the use of steroids [35]. However, more trials are needed before its efficacy can be established.

### Vaccines

At present, no vaccine is available for the treatment of COVID-19. However, much research is in progress to develop an effective vaccine against this virus. According to the World Health Organization, 29 candidate vaccines are in clinical evaluation, and 138 are in preclinical evaluation. India is developing three vaccines that are under clinical trial and listed in Table-1, along with other clinical candidate vaccines [67].

**Table-1:** Candidate vaccines in clinical trial.

S. No.	Developer	Country	Vaccine platform	Type of candidate vaccine	Number of doses	The current stage of clinical evaluation
1	University of Oxford/AstraZeneca	UK	Non-replicating viral vector	ChAdOx1-S	3	Phase 3
2	Sinovac65	China	Inactivated	Inactivated	2	Phase 3
3	Wuhan Institute of Biological 65`Products/Sinopharm	China	Inactivated	Inactivated	2	Phase 3
4	Beijing Institute of Biological Products/Sinopharm	China	Inactivated	Inactivated	2	Phase 3
5	Moderna/NIAID	USA	RNA	LNP-encapsulated mRNA	2	Phase 3
6	BioNTech/Fosun Pharma/Pfizer	German/China/USA	RNA	3 LNP-mRNAs	2	Phase 3
7	CanSino Biological Inc./Beijing Institute of Biotechnology	China	Non-replicating viral vector	Adenovirus Type 5 vector	1	Phase 2
8	Anhui Zhifei Longcom Biopharmaceutical/Institute of Microbiology, Chinese Academy of Science	China	Protein Subunit	Adjuvanted recombinant protein (RBD-Dimer)	2 or 3	Phase 2
9	Institute of Medical Biology, Chinese Academy of Medical Sciences	China	Inactivated	Inactivated	2	Phase 1/2
10	Inovio Pharmaceuticals/International Vaccine Institute	USA/South Korea	DNA	DNA plasmid vaccine with electroporation	2	Phase 1/2
11	Osaka University/AnGes/Takara Bio	Japan	DNA	DNA plasmid vaccine + Adjuvant	2	Phase 1/2
12	Cadila Healthcare Limited	India	DNA	DNA plasmid vaccine	3	Phase 1/2
13	Genexine Consortium	Republic Of Korea	DNA	DNA Vaccine (GX-19)	2	Phase 1/2
14	Bharat Biotech	India	Inactivated	Whole-Virion Inactivated	2	Phase 1/2
15	Janssen Pharmaceutical Companies	USA	Non-replicating viral vector	Ad26COVS1	2	Phase 1/2
16	Novavax	Sweden	Protein Subunit	Full length recombinant SARS-CoV2 glycoprotein nanoparticle vaccine adjuvanted with Matrix M	2	Phase 1/2
17	Kentucky Bioprocessing, Inc	USA	Protein Subunit	RBD-based	2	Phase 1/2
18	Arcturus/Duke-NUS	USA/ Singapore	RNA	mRNA		Phase 1/2
19	Gamaleya Research Institute	Russia	Non-Replicating Viral Vector	Adeno-based	1	Phase 1
20	ReiThera/LEUKOCARE/Univercells N	Italy/ Germany/Belgium	Non-Replicating Viral Vector	Replication defective Simian Adenovirus (GRAd) encoding S	1	Phase 1
21	Clover Biopharmaceuticals Inc./GSK/Dynavax	China/UK/USA	Protein Subunit	Native like Trimeric subunit Spike Protein vaccine	2	Phase 1
22	Vaxine Pty Ltd/Medytox	Australia/South Korea	Protein Subunit	Recombinant spike protein with Advax™ adjuvant	1	Phase 1
23	University of Queensland/CSL/Seqirus	Australia	Protein Subunit	Molecular clamp stabilized Spike protein with MF59 adjuvant	2	Phase 1
24	Institut Pasteur/Themis/Univ. of Pittsburg CVR/Merck Sharp and Dohme	France/USA	Replicating Viral Vector	Measles-vector based	1 or 2	Phase 1
25	Imperial College London	UK	RNA	LNP-nCoVsaRNA	2	Phase 1
26	Curevac	Germany	RNA	mRNA	2	Phase 1
27	People’s Liberation Army Academy of Military Sciences/Walvax Biote	China	RNA	mRNA	2	Phase 1
28	Medicago Inc.	Canada	VLP	Plant-derived VLP adjuvanted with GSK or Dynavax adjs.	2	Phase 1
29	Medigen Vaccine Biologics Corporation/NIAID/Dynavax	Taiwan/USA	Protein Subunit	S-2P protein + CpG 1018	2	Phase 1

SARS-CoV2=Severe acute respiratory syndrome coronavirus-2

## Conclusion

The SARS-CoV-2 has existed in the world for over 6 months. During this short duration, it has spread across 216 Countries/Territories/Areas of the world. Even developed countries could not prevent themselves from the damages caused by this virus. Several measures, such as lockdown, random testing, and various researches related to therapies, have been taken to contain the spread of this disease. Some drugs have shown promising results to fight against the SARS-CoV-2, which results in a higher recovery rate. As there are no specific drugs or vaccines are available, it is impossible to predict the end of this pandemic. However, several vaccines are under clinical trials, but it will take a minimum of 6-8 months before it reaches the general population. Moreover, viruses tend to mutate, so we can never be confident that vaccines will work. Therefore, we will have to rely on preventive measures such as frequent hand washing, use of masks, restricted traveling, personal hygiene, and healthy eating to keep ourselves COVID- 19 free until the proper and specific treatment is available.

## Authors’ Contributions

ST drafted and revised the manuscript. MMT read, edited and approved the final manuscript. Both authors read and approved the final manuscript.

## References

[1] Huang C, Wang Y, Li X, Ren L, Zhao J, Hu Y, Zhang L, Fan G, Xu J, Gu X, Cheng Z, Yu T, Xia J, Wei Y, Wu W, Xie X, Yin W, Li H, Liu M, Xiao Y, Gao H, Guo L, Xie J, Wang G, Jiang R, Gao Z, Jin Q, Wang J, Cao B (2020). Clinical features of patients infected with 2019 novel Coronavirus in Wuhan, China. Lancet.

[2] Chen N, Zhou M, Dong X, Qu J, Gong F, Han Y, Qiu Y, Wang J, Liu Y, Wei Y, Xia J, Yu T, Zhang X, Zhang L (2020). Epidemiological and clinical characteristics of 99 cases of 2019 novel Coronavirus pneumonia in Wuhan, China:A descriptive study. Lancet.

[3] Wang D, Hu B, Hu C, Zhu F, Liu X, Zhang J, Wang B, Xiang H, Cheng Z, Xiong Y, Zhao Y, Li Y, Wang X, Peng Z (2020). Clinical characteristics of 138 hospitalized patients with 2019 novel Coronavirus-infected pneumonia in Wuhan, China. JAMA.

[4] International Committee on Taxonomy of Virus (2020). Naming the 2019 Coronavirus.

[5] (2020). Notice of the National Health Commission of the People's Republic of China on Revising the English name of Novel Coronavirus Pneumonia.

[6] Zaki A.M, van Boheemen S, Bestebroer T.M, Osterhaus A.D, Fouchier R.A (2012). Isolation of a novel Coronavirus from a man with pneumonia in Saudi Arabia. N. Engl. J Med.

[7] Chan-Yeung M, Xu R.H (2003). SARS:Epidemiology. Respirology.

[8] World Health Organization (2020). Situation Report-208.

[9] Ministry of Health and Family Welfare (2020). Update on Novel Coronavirus:One Positive Case Reported in Kerala.

[10] Ministry of Health and Family Welfare (2020). One of the Lowest Globally, India's Case Fatality Rate Below 2% and Sliding.

[11] Shereen M.A, Khan S, Kazmi A, Bashir N, Siddique R (2020). COVID-19 infection:Origin, transmission, and characteristics of human Coronaviruses. J. Adv. Res.

[12] Fehr A.R, Perlman S (2015). Coronaviruses:An overview of their replication and pathogenesis. Methods Mol. Biol.

[13] Zhu N, Dingyu Z, Wenling W, Xingwang L, Bo Y, Jingdong S, Xiang Z, Baoying H, Weifeng S, Roujian L, Peihua N, Faxian Z, Xuejun M, Dayan W, Wenbo X, Guizhen W, George F.G, Wenjie T (2019). A novel Coronavirus from patients with pneumonia in China, 2019. N. Engl. J. Med.

[14] Perlman S (2020). Another decade, another Coronavirus. N. Engl. J. Med.

[15] Xu X, Chen P, Wang J, Zhong W, Feng J, Zhou H, Li X, Hao P (2020). Evolution of the novel Coronavirus from the ongoing Wuhan outbreak and modeling of its spike protein for risk of human transmission. Sci. China Life Sci.

[16] Wu F, Zhao S, Yu B, Chen Y.M, Wang W, Song Z.G, Hu Y, Tao Z.W, Tian J.H, Pei Y.Y, Yuan M.L, Zhang Y.L, Dai F.H, Liu Y, Wang Q.M, Zheng J.J, Xu L, Holmes E.C, Zhang Y.Z (2020). A new Coronavirus associated with human respiratory disease in China. Nature.

[17] Zhou P, Yang X.L, Wang X.G, Hu B, Zhang L, Zhang W, Si H.R, Zhu Y, Li B, Huang C.L, Chen H.D, Chen J, Luo Y, Guo H, Jiang R.D, Liu M.Q, Chen Y, Shen X.R, Wang X, Zheng X.S, Zhao K, Chen Q.J, Deng F, Liu L.L, Yan B, Zhan F.X, Wang Y.Y, Xiao G.F, Shi Z.L (2020). A pneumonia outbreak associated with a new Coronavirus of probable bat origin. Nature.

[18] Lam T.T.Y, Shum M.H.H, Zhu H.C, Tong Y.G, Ni X.B, Liao Y.S, Wei W, Cheung W.Y.M, Li W.J, Li L.F, Leung G.M, Holmes E.C, Hu Y.L, Guan Y (2020). Identification of 2019-nCoV Related Corona Viruses in Malayan Pangolins in Southern China, Bio Rxiv.

[19] Wu Y.C, Chen C.S, Chan Y.J (2020). The outbreak of COVID-19:An overview. J. Chin. Med. Assoc.

[20] Xinhua (2020). China's CDC Detects a Large Number of New Coronavirus in the South China Seafood Market in Wuhan.

[21] Yu W.B, Tang G.D, Zhang L.C (2020). Decoding the evolution and transmissions of the novel pneumonia Coronavirus (SARS-CoV-2/HCoV-19) using whole genomic data. Zool. Res.

[22] Ministry of Health and Family Welfare (2020). The Outbreak of Novel Corona Virus in China:Actions Taken by the Health Ministry.

[23] Prime Minister's Office (2020). PM Calls for Complete Lockdown of the Entire Nation for 21 Days:PM Addresses the Nation on COVID-19.

[24] Wu A, Peng Y, Huang B, Ding X, Wang X, Niu P, Meng J, Zhu Z, Zhang Z, Wang J, Sheng J, Quan L, Xia Z, Tan W, Cheng G, Jiang T (2020). Genome composition and divergence of the novel Coronavirus (2019-nCoV) originating in China. Cell Host Microbe.

[25] Devendran R, Kumar M, Chakraborty S (2020). Genome analysis of SARS-CoV-2 isolates occurring in India:Present scenario. Indian J. Public Health.

[26] Central for Diseases Control and Prevention (2020). How COVID-19 Spreads.

[27] World Health Organization (2020). Situation Reports.

[28] Lu C.W, Liu X.F, Jia Z.F (2020). 2019-nCoV transmission through the ocular surface must not be ignored. Lancet.

[29] Chen H, Guo J, Wang C, Luo F, Yu X, Zhang W, Li J, Zhao D, Xu D, Gong Q, Liao J, Yang H, Hou W, Zhang Y (2020). Clinical characteristics and intrauterine vertical transmission potential of COVID-19 infection in nine pregnant women:A retrospective review of medical records. Lancet.

[30] Li Q, Guan X, Wu P, Wang X, Zhou L, Tong Y, Ren R, Leung K.S.M, Lau E.H.Y, Wong J.Y, Xing X, Xiang N, Wu Y, Li C, Chen Q, Li D, Liu T, Zhao J, Liu M, Tu W, Chen C, Jin L, Yang R, Wang Q, Zhou S, Wang R, Liu H, Luo Y, Liu Y, Shao G, Li H, Tao Z, Yang Y, Deng Z, Liu B, Ma Z, Zhang Y, Shi G, Lam T.T.Y, Wu J.T, Gao G.F, Cowling B.J, Yang B, Leung G.M, Feng Z (2020). Early transmission dynamics in Wuhan, China, of novel Coronavirus-infected pneumonia. N. Engl. J. Med.

[31] Medical Expert Group of Tongji Hospital. Quick Guide to the Diagnosis and Treatment of Pneumonia for Novel Coronavirus Infections. Herald of Medicine (2020). Medical Expert Group of Tongji Hospital, Huazhong University of Science and Technology.

[32] Liu T, Hu J, Xiao J, He G, Kang M, Rong Z, Lin L, Zhong H, Huang Q, Deng A, Zeng W, Tan X, Zeng S, Zhu Z, Li J, Gong D, Wan D, Chen S, Guo L, Li Y, Sun L, Liang W, Song T, He J, Ma W (2020). Time-Varying Transmission Dynamics of Novel Corona Virus Pneumonia in China. Bio Rxiv.

[33] Adhikari S.P, Meng S, Wu Y.J, Mao Y.P, Ye R.X, Wang Q.Z, Sun C, Sylvia S, Rozelle S, Raat H, Zhou H (2020). Epidemiology, causes, clinical manifestation and diagnosis, prevention and control of Coronavirus disease (COVID-19) during the early outbreak period:A scoping review. Infect. Dis. Poverty.

[34] World Health Organization (2020). Coronavirus.

[35] Government of India Ministry of Health and Family Welfare Directorate General of Health Services (EMR Division) (2020). Clinical Management of COVID-19 Protocol.

[36] Indian Council of Medical Research (2020). Newer Additional Strategies for COVID-19 Testing.

[37] Tang J.W, Tambyah P.A, Hui D.S.C (2020). Emergence of a novel Coronavirus causing respiratory illness from Wuhan, China. J. Infect.

[38] Habibzadeh P, Stoneman E.K (2020). The novel Coronavirus:A bird's eye view. Int. J. Occup. Environ. Med.

[39] World Health Organization (2020). Global Surveillance for COVID-19 Caused by Human Infection with COVID-19 Virus.

[40] NIH (2020). Coronavirus Disease 2019 (COVID-19) Treatment Guidelines.

[41] Wang M, Cao R, Zhang L, Yang X, Liu J, Xu M, Shi Z, Hu Z, Zhong W, Xiao G (2020). Remdesivir and chloroquine effectively inhibit the recently emerged novel Coronavirus (2019-nCoV) *in vitro*. Cell Res.

[42] Gao J, Tian Z, Yang X (2020). Breakthrough:Chloroquine phosphate has shown apparent efficacy in treatment of COVID-19 associated pneumonia in clinical studies. Biosci Trends.

[43] (2020). Chinese Clinical Trial Registry.

[44] Gautret P, lagier J.C, Parola P, Hoang V.T, Medded L, Mailhe M, Doudier B, Courjon J, Giordanengo V, Esteves V.V, Tissot-Dupont H, Honore S, Colson P, Chabriere E, La Scola B, Rolain J.M, Brouqui P, Raoult D (2020). Hydroxychloroquine and Azithromycin as a Treatment of COVID-19:Preliminary Results of an Open-Label Non-Randomized Clinical Trial, Med Rxiv.

[45] Lai S.T (2005). Treatment of severe acute respiratory syndrome. Eur J Clin . Microbiol. Infect. Dis.

[46] Soo Y, Cheng Y, Wong R, Hui D.S, Lee C.K, Tsang K.K, Ng M.H, Chan P, Cheng G, Sung J.J (2004). Retrospective comparison of convalescent plasma with continuing high-dose clinical microbiology and infection. Eur J Clin. Microbiol. Infect. Dis.

[47] Cheng Y, Wong R, Soo Y.O, Wong W.S, Lee C.K, Ng M.H, Chan P, Wong K.C, Leung C.B, Cheng G (2005). Use of convalescent plasma therapy in SARS patients in Hong Kong. Eur. J .Clin. Microbiol. Infect. Dis.

[48] Derebail V.K, Falk R.J (2020). ANCA-associated vasculitis-refining therapy with plasma exchange and glucocorticoids. N Engl. J. Med.

[49] Kakodkar P, Kaka N, Baig M.N (2020). A comprehensive literature review on the clinical presentation and management of the pandemic Coronavirus disease 2019 (COVID-19). Cureus.

[50] Klimas J, Olvedy M, Ochodnicka-Mackovicova K, Kruzliak P, Cacanyiova S, Kristek F, Krenek P, Ochodnicky P (2015). Perinatally administered losartan augments renal ACE2 expression but not cardiac or renal mas receptor in spontaneously hypertensive rats. J Cell Mol. Med.

[51] Sheahan T.P, Sims A.C, Leist S.R, Schäfer A, Won J, Brown A.J, Montgomery S.A, Hogg A, Babusis D, Clarke M.O, Spahn J.E, Bauer L, Sellers S, Porter D, Feng J.Y, Cihlar T, Jordan R, Denison M.R, Baric R.S (2020). Comparative therapeutic efficacy of remdesivir and combination lopinavir, ritonavir, and interferon-beta against MERS-CoV. Nat. Commun.

[52] Holshue M.L, DeBolt C, Lindquist S, Lofy K.H, Wiesman J, Bruce H, Spitters C, Ericson K, Wilkerson S, Tural A, Diaz G, Cohn A, Fox L, Patel A, Gerber S.I, Kim L, Tong S, Lu X, Lindstrom S, Pallansch M.A, Weldon W.C, Biggs H.M, Uyeki T.M, Pillai S.K (2020). First case of 2019 novel Coronavirus in the United States. N. Engl. J. Med.

[53] The National Heart Lung and Blood Institute Acute Respiratory Distress Syndrome (ARDS) Clinical Trials Network. Efficacy and safety of corticosteroids for persistent acute respiratory distress syndrome (2006). N. Engl. J. Med.

[54] Choy K.T, Wong A.Y, Kaewpreedee P, Sia S.F, Chen D, Hui K, Chu D, Chan M, Cheung P.P, Huang X, Peiris M, Yen H.L (2020). Remdesivir, lopinavir, emetine, and homoharringtonine inhibit SARS-CoV-2 replication *in vitro*. Antiviral Res.

[55] Samsonraj R.M, Raghunath M, Nurcombe V, Hui J.H, van Wijnen A.J, Cool S.M (2017). Concise review:Multifaceted characterization of human mesenchymal stem cells for use in regenerative medicine. Stem Cells Transl Med.

[56] Taghavi-Farahabadi M, Mahmoudi M, Soudi S, Hashemi S.M (2020). Hypothesis for the management and treatment of the COVID-19-induced acute respiratory distress syndrome and lung injury using mesenchymal stem cell-derived exosomes. Med. Hypotheses.

[57] Li N, Hua J (2017). Interactions between mesenchymal stem cells and the immune system. Cell Mol. Life Sci.

[58] Food and Drug Administration (2019). FDA Warns about Stem Cell Therapies.

[59] Le R.Q, Li L, Yuan W, Shord S.S, Nie L, Habtemariam B.A, Przepiorka D, Farrell A.T, Pazdur R (2018). FDA approval summary:Tocilizumab for treatment of chimeric antigen receptor T cell-induced severe or life-threatening cytokine release syndrome. Oncologist.

[60] Xu Z, Shi L, Wang Y, Zhang J, Huang L, Zhang C, Liu S, Zhao P, Liu H, Zhu L, Tai Y, Bai C, Gao T, Song J, Xia P, Dong J, Zhao J, Wang F.S (2020). Pathological findings of COVID-19 associated with acute respiratory distress syndrome. Lancet Respir. Med.

[61] Xu X, Han M, Li T, Sun W, Wang D, Fu B, Zhou Y, Zheng X, Yang Y, Li X, Zhang X, Pan A, Wei H (2020). Effective treatment of severe COVID-19 patients with tocilizumab. Proc. Natl. Acad. Sci. U. S. A.

[62] Luo P, Liu Y, Qiu L, Liu X, Liu D, Li J (2020). Tocilizumab treatment in COVID-19:A single center experience. J. Med. Virol.

[63] Campochiaro C, Della-Torre E, Cavalli G, de Luca G, Ripa M, Boffini N, Tomelleri A, Baldissera E, Rovere-Querini P, Ruggeri A, Monti G, de Cobelli F, Zangrillo A, Tresoldi M, Castagna A (2020). Dagna L and TOCI-RAF Study Group. Efficacy and safety of tocilizumab in severe COVID-19 patients:A single-centre retrospective cohort study. Eur. J. Intern. Med.

[64] Morena V, Milazzo L, Oreni L, Bestetti G, Fossali T, Bassoli C, Torre A, Cossu M.V, Minari C, Ballone E, Perotti A, Mileto D, Niero F, Merli S, Foschi A, Vimercati S, Rizzardini G, Sollima S, Bradanini L, Galimberti L, Colombo R, Micheli V, Negri C, Ridolfo A.L, Meroni L, Galli M, Antinori S, Corbellino M (2020). Off-label use of tocilizumab for the treatment of SARS-CoV-2 pneumonia in Milan, Italy. Eur. J. Intern. Med.

[65] Toniati P, Piva S, Cattalini M, Garrafa E, Regola F, Castelli F, Franceschini F, Airò P, Bazzani C, Beindorf E.A, Berlendis M, Bezzi M, Bossini N, Castellano M, Cattaneo S, Cavazzana I, Contessi G.B, Crippa M, Delbarba A, de Peri E, Faletti A, Filippini M, Filippini M, Frassi M, Gaggiotti M, Gorla R, Lanspa M, Lorenzotti S, Marino R, Maroldi R, Metra M, Matteelli A, Modina D, Moioli G, Montani G, Muiesan M.L, Odolini S, Peli E, Pesenti S, Pezzoli M.C, Pirola I, Pozzi A, Proto A, Rasulo F.A, Renisi G, Ricci C, Rizzoni D, Romanelli G, Rossi M, Salvetti M, Scolari F, Signorini L, Taglietti M, Tomasoni G, Tomasoni L.R, Turla F, Valsecchi A, Zani D, Zuccalà F, Zunica F, Focà E, Andreoli L, Latronico N (2020). Tocilizumab for the treatment of severe COVID-19 pneumonia with hyperinflammatory syndrome and acute respiratory failure:A single center study of 100 patients in Brescia, Italy. Autoimmun. Rev.

[66] Biran N, Ip A, Ahn J, Go R.C, Wang S, Mathura S, Sinclaire B.A, Bednarz U, Marafelias M, Hansen E, Siegel D.S, Goy A.H, Pecora A.L, Sawczuk I.S, Koniaris L.S, Simwenyi M, Varga D.W, Tank L.K, Stein A.A, Allusson V, Lin G.S, Oser W.F, Tuma R.A, Reichman J, Brusco L, Carpenter K.L, Costanzo E.J, Vivona V, Goldberg S.L (2020). Tocilizumab among patients with COVID-19 in the intensive care unit:A multicentre observational study. Lancet Rheumatol.

[67] World Health Organization (2020). DRAFT Landscape of COVID-19 Candidate Vaccines.

